# Predictors of Ibandronate Efficacy for the Management of Osteoporosis: A Meta-Regression Analysis

**DOI:** 10.1371/journal.pone.0150203

**Published:** 2016-03-01

**Authors:** Zeren Ma, Yong Li, Ming Zhou, Kedi Huang, Hejun Hu, Xiaoping Liu, Xiaosheng Xu

**Affiliations:** 1 Spinal and Joint Unit, Department of Orthopedics, Nanchang Hongdu Hospital of Traditional Chinese Medicine, Nanchang, Jiangxi 330006, China; 2 Department of Orthopaedics, Shanxi Province People’s Hospital, Xi’an 710068, China; Van Andel Institute, UNITED STATES

## Abstract

**Background:**

Aim of the present study was to identify the predictors of ibandronate efficacy in subjects with osteoporosis or decreased bone mineral density (BMD).

**Method:**

Several electronic databases were searched by using specific keywords for the acquisition of research articles reporting the efficacy of ibandronate in subjects with osteoporosis or decreased BMD. Metaregression analyses were carried out by using changes in the BMD of lumbar spine and total hip following ibandronate treatment as dependent (outcome) variables against several independent (explanatory) variables.

**Results:**

Data were extracted from 34 studies (11,090 ibandronate treated subjects) which fulfilled eligibility criteria. A history of previous fracture/s was reported by 46% of these subjects. In overall population, longer treatment duration from 1 to 5 years, increasing age, history of previous fractures, lower baseline T score, and higher baseline levels of C-terminal telopeptide of type 1 collagen (CTX) predicted higher ibandronate efficacy in improving BMD of the lumbar spine as well as of the total hip. Lower baseline levels of vitamin D and higher baseline levels of bone specific alkaline phosphatase (BSAP) predicted higher efficacy of ibandronate for lumbar spine only. In postmenopausal women with osteoporosis or decreased BMD, in addition to above-mentioned predictors, better efficacy of ibandronate was also associated with increasing time since menopause for both lumbar spine and total hip and lower body weight for lumbar spine only.

**Conclusion:**

Longer treatment duration from 1 to 5 years, increasing age, lower baseline T scores, and higher serum CTX levels are identified as the predictors of better efficacy of ibandronate in the study subjects with osteoporosis or decreased BMD.

## Introduction

Osteoporosis is a disease in which the bone mineral density (BMD) and the quality of bone are reduced that predisposes individuals to a higher risk of low-trauma factures [1]. Annual incidence of osteoporotic fractures worldwide is estimated at about9 millions of which 75–80% fractures are sustained by 200 million women suffering from osteoporosis [2]. This poses a significant global public health concern with socioeconomic implications. It is estimated that the incidence of hip fractures will increase up to 240% in women and 310% in men by the year 2050 [3,4].

Osteoporosis cause significant morbidity and mortality in elderly. It can cause kyphosis, restrictive lung disease, abdominal distension, and height loss. Osteoporotic fractures are more common in vertebrae, hip and wrist [5]. Hip fractures are associated with significantly higher mortality rates and most of the patients die within 3–6 months after the event. Among the survivors majority of the patients face significantly compromised performance in their activities of daily living [6–8]. Osteoporotic fracture risk increases with age; whereas 10-year fracture risk at the age of 50 years is 9.8% in women and 7.1% in men, at the age of 80, it is 21.7% in women and 8% in men [9].

Of the multiple therapeutic options available for the management of osteoporosis, bisphosphonates act to reduce osteoclast mediated bone resorption [10,11] by inducing osteoclast suppression and apoptosis [12]. Aminobisphosphonates including alendronate, ibandronate, risedronate and zoledronate are considered more efficacious than non-nitrogenous bisphosphonates. Because of the poor bioaccessibility of orally taken bisphosphonates (less than 1% absorption in the gut) and inadequate patient compliance [13], bisphosphonates may also be infused via the intravenous route which enhances their bioavailability at much lower frequency of administration.

Among the bisphosphonates, ibandronate offers relatively flexible dosing formulations and administration schedule. A number of trials have examined the efficacy of ibandronate in subjects with osteoporosis or decreased BMD [14–58] and majority of these studies found ibandronate significantly efficacious in improving BMD which is also reported by recent meta-analyses [59,60]. Identifying predictors of ibandronate efficacy can help in decision making as multiple options are available for treating osteoporosis which has a multifactorial etiology. We have used data from 34 studies for carrying out meta-regression analyses in order to identify the predictors of ibandronate efficacy in improving BMD of lumbar spine and total hip in subjects with osteoporosis or decreased BMD.

## Methods

### Literature search strategy

Relevant studies were identified after a comprehensive literature search in multiple electronic databases including EMBASE, Google Scholar, OVID SP, PubMed and Web of Science. The major medical subject headings (MeSH) and keywords were used in different combinations. For primary search, [ibandronate bone mineral density osteoporosis] combination was used. For secondary searches, various combinations including [ibandronate postmenopausal osteoporosis], [ibandronate bone mineral density osteoporosis lumbar spine], [ibandronate bone mineral density osteoporosis hip], [ibandronate bone mineral density postmenopausal osteoporosis (PMO)], [ibandronate osteoporosis fractures], [ibandronate osteoporosis bone resorption], [ibandronate osteoporosis osteocalcin], [ibandronate osteoporosis sclerostin], [ibandronate osteoporosis C-terminal telopeptide of type 1 collagen (CTX)], [ibandronate osteoporosis bone specific alkaline phosphatase (BSAP)], [ibandronate osteoporosis procollagen type I N-terminal propeptide (PINP)], [ibandronate osteoporosis parathyroid hormone (PTH)], and [ibandronate osteoporosis vitamin D] were used. Same strategy was used for each database. The search encompassed original research papers published before February 2015. Bibliographies of important relevant research articles were manually searched. This study does not involve ethical review.

### Inclusion and exclusion criteria

The inclusion criterion was—trials evaluating the efficacy of ibandronate by treating subjects (individuals with osteoporosis or decreased BMD) for one or more years and measured baseline, later stage/s and endpoint BMD of lumbar spine and/or hip. The study/studies was/were excluded if it/these used ibandronate for the purpose other than skeletal improvement; utilized ibandronate in combination with other therapeutic regimens; provided relevant but inadequate information regarding the measures of data spread or the variables of interests; or studied persistence/adherence data only.

### Data extraction and statistical analysis

Information on demographic characteristics of the study subjects, osteoporotic condition, trial endpoints, outcomes, and baseline values of related serological markers and other clinical characteristics were extracted from each study report/s and tabulated in Microsoft Excel spreadsheets. The metaregression analyses were stratified first by lumbar spine and total hip BMD for overall population of this study and then by the postmenopausal women with osteoporosis or decreased BMD and non-PMO subjects with osteoporosis or decreased BMD. For each of the dependent variables (percent changes from baseline in the BMD of lumbar spine or total hip), we tested several explanatory (independent) variables including treatment duration, number of ibandronate treated patients, gender, age, weight, height, body mass index (BMI) of the subjects, history of previous fractures, time since menopause, baseline lumbar spine / total hip T scores, and baseline 25-OH vitamin D, PTH, osteocalcin, CTX, PINP, BSAP, calcium, and phosphate levels in the blood. After calculating the percent changes in the BMD of lumbar spine and total hip for each of the included studies, metaregression analyses were carried out with STATA software (Version 12; College station, Texas) under random effects model using restricted maximum likelihood method. A P<0.1 was considered to be significant. Statistical indices for heterogeneity assessment were tau^2^ and I^2^.

## Results

**Fig 1:**
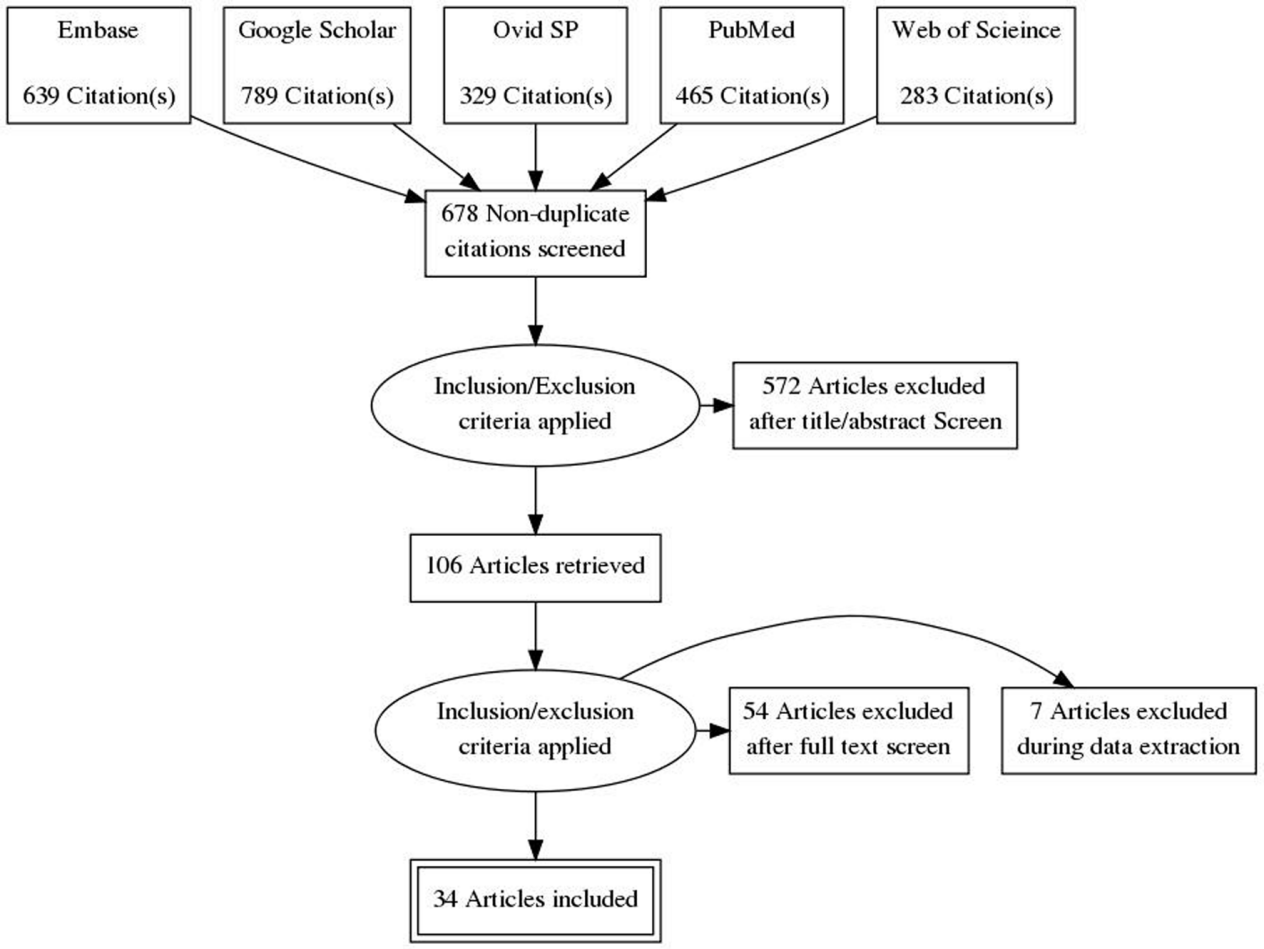
Flowchart of study screening and selection process.

Thirty four studies [14–58] including 28 randomized controlled, 1 non-randomized controlled [15], 4 prospective observational [26,30,36,57], and 1 retrospective [50] studies fulfilled eligibility criteria. A flowchart of study screening and selection process is presented in Fig 1. Overall population of this meta-analysis was 11,090 ibandronate treated subjects of which 7,531 were administered ibandronate orally and 3559 intravenously.

Age, height, weight and BMI of the patients as mean and standard deviation were 62.4 ± 7.6 years, 159 ± 6.7 centimeters, 64.7 ± 12 kilograms, and 25.3 ± 4.4 kg/m^2^, respectively. Duration of ibandronate treatment in these trials was 1.9 ± 1.1 (1–5) years. A history of previous fractures was reported by 46% of the study subjects. In postmenopausal women with osteoporosis or decreased BMD, time since menopause was 15.4 ± 7 years. Baseline values of related serum markers were: Vitamin D 25OH (30 ± 12 ng/ml), PTH (50 ± 25 pg/ml), osteocalcin (24 ± 10 ng/ml), CTX (0.4 ± 0.3 ng/ml), PINP (50 ± 31 ng/ml), BSAP (59 ± 20 U/l), calcium (9.4 ± 0.5 mg/dl), and serum phosphate (3.7 ± 0.6 mg/dl).

**Fig 2:**
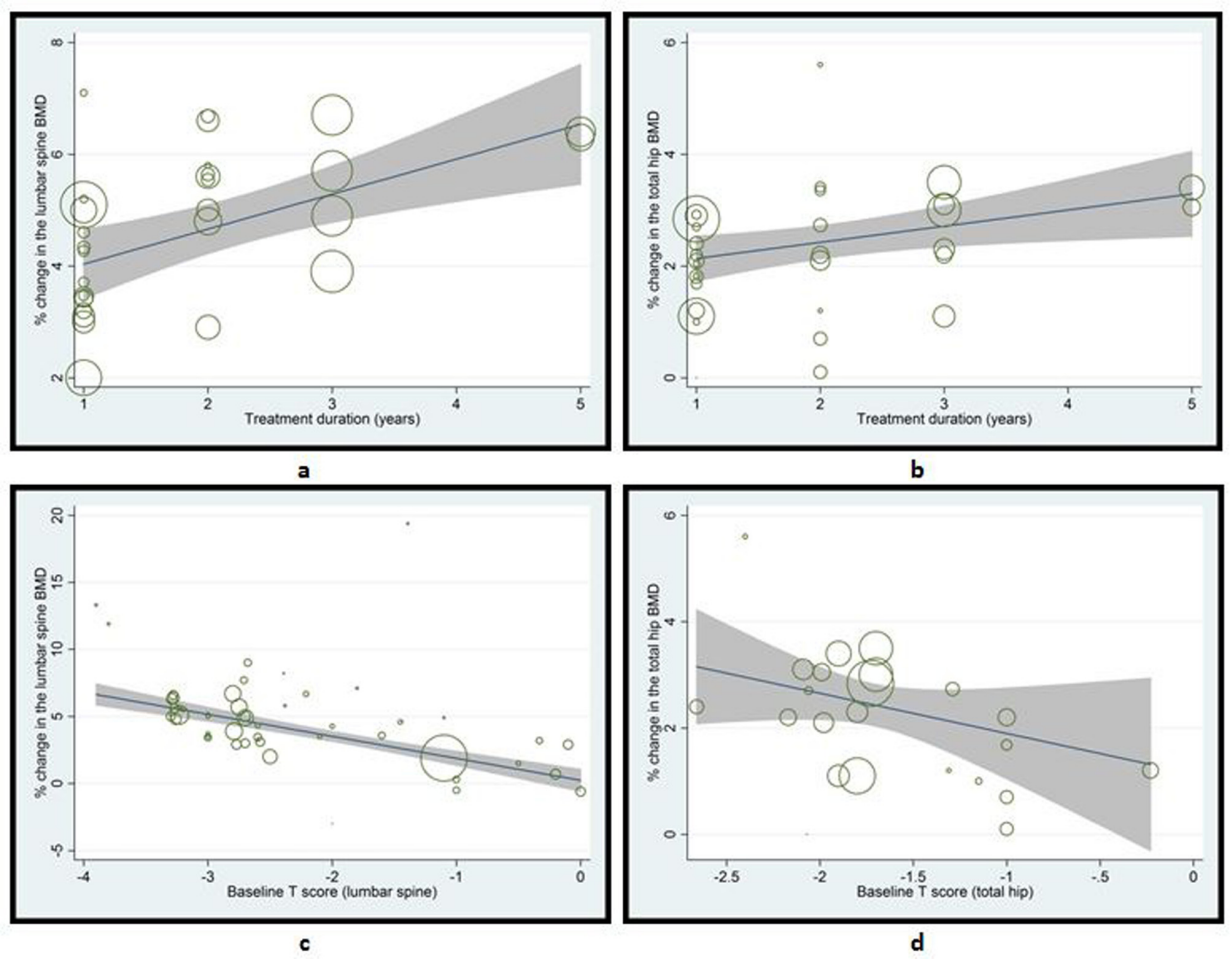
Scatterplots showing the relationships between ibandronate efficacy in terms of percent change in BMD of lumbar spine (a & c) or total hip (b & d) and treatment duration (a & b) or baseline T scores (c & d). See Table 1 for corresponding values.

**Fig 3:**
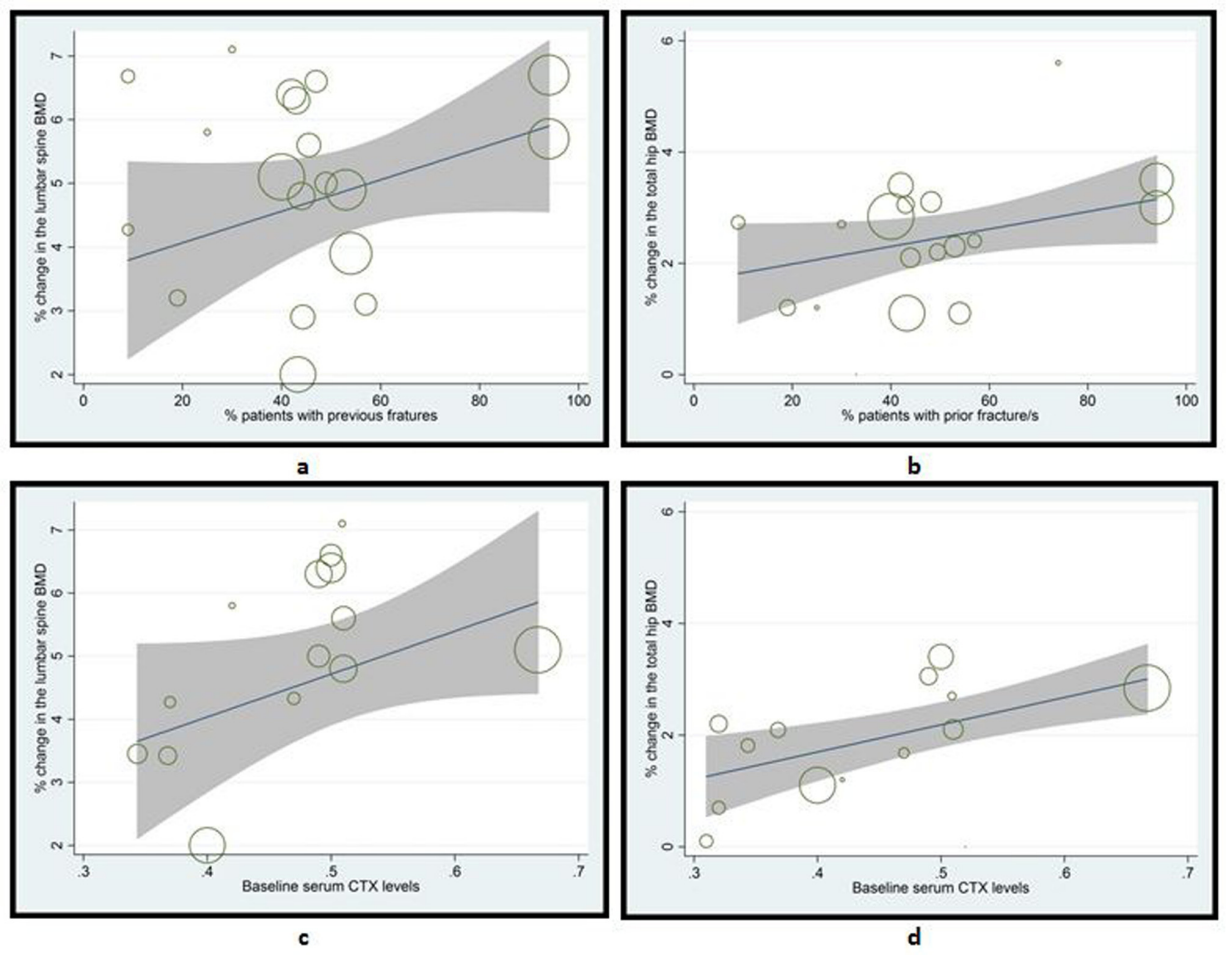
Scatterplots showing the relationships between ibandronate efficacy in terms of percent change in BMD of lumbar spine (a & c) or total hip (b & d) and percent patients with a history of previous fractures (a & b) or baseline serum CTX levels (c & d). See Table 1 for corresponding values.

Based on the statistical significance in the metaregression analyses, several predictors of the efficacy of ibandronate in improving BMD of the lumbar spine in subjects with osteoporosis or decreased BMD (all conditions) were identified. Longer treatment duration from 1 to 5 years, increasing age, lower body weight, history of previous fractures, lower baseline T score of lumbar spine, lower baseline levels of 25-OH vitamin D, and higher baseline levels of serum BSAP and CTX were significantly associated with higher efficacy of ibandronate (Table 1; Figs 2 and 3). On the other hand, number of participants, gender, height, BMI, baseline serum levels of PTH, PINP, osteocalcin, calcium and phosphate did not show any significant relationship with the efficacy of ibandronate in improving lumbar spine BMD in the overall population of this meta-analysis.

**Table 1:** Predictors of the efficacy of ibandronate in improving BMD in osteoporosis patients.

Change in lumbar spine BMD	No. of datasets	tau^2^	Coefficient [95% CI]	P
**Lumbar spine**				
Treatment duration	50	9.22	1.086 [0.141, 2.032]	**0.025**
Number of participants	50	10.36	0.00057 [−0.003, 0.004]	0.752
Gender	50	10.29	0.0063 [−0.025, 0.038]	0.683
Age	49	10.07	0.215 [0.101, 0.327]	**0.001**
Body weight	34	5.42	−0.183 [−0.335, -0.031]	**0.020**
Body height	30	4.903	0.0063 [−0.237, 0.249]	0.958
Body mass index	35	4.77	−0.353 [−0.907, 0.202]	0.205
History of fractures	27	4.504	0.0382 [0.0003, 0.078]	**0.053**
Baseline T score (lumbar spine)	50	7.886	−1.432 [−2.296, −0.569]	**0.002**
Baseline serum vitamin D 25OH	31	3.104	−0.252 [−0.365, −0.141]	**0.0001**
Baseline serum PTH	16	5.245	−0.027 [−0.068, 0.014]	0.191
Baseline serum osteocalcin	21	5.04	−0.089 [−0.245, 0.066]	0.241
Baseline serum BSAP	19	10.75	0.0565 [0.0115, 0.1016]	**0.017**
Baseline serum CTX	23	1.924	17.947 [11.33, 24.57]	**0.0001**
Baseline serum PINP	5	1.454	−0.0686 [−0.228, 0.091]	0.264
Baseline serum calcium	9	0.432	−1.46 [−8.884, 5.964]	0.656
Baseline serum phosphate	5	5.079	−2.982 [−12.29, 6.32]	0.383
**Total hip**				
Treatment duration	32	0.718	0.274 [−0.028, 0.577]	**0.074**
Number of participants	32	0.811	0.0004 [−0.0007, 0.0015]	0.461
Gender	32	0.702	−0.0088 [−0.022, 0.004]	0.163
Age	32	0.665	0.0609 [−0.002, 0.124]	**0.057**
Body weight	22	0.292	0.0106 [−0.044, 0.065]	0.692
Body height	22	0.289	−0.0121 [−0.093, 0.069]	0.759
Body mass index	24	0.598	−0.1143 [−0.373, 0.144]	0.370
History of fractures	18	0.68	0.0176 [−0.0032, 0.0385]	**0.092**
Baseline T score (total hip)	23	0.747	−1.068 [−1.818, −0.319]	**0.007**
Baseline serum vitamin D O,25	22	0.781	−0.422 [−0.120, 0.036]	0.272
Baseline serum PTH	10	0.968	0.0087 [−0.039, 0.057]	0.689
Baseline serum osteocalcin	16	0.955	0.0182 [−0.069, 0.106]	0.662
Baseline serum BSAP	14	0.917	0.0088 [−0.009, 0.027]	0.309
Baseline serum CTX	14	0.5	5.988 [1.483, 10.494]	**0.013**
Baseline serum PINP	4	1.09	−0.159 [−0.499, 0.182]	0.183
Baseline serum calcium	7	1.314	−3.266 [−11.32, 4.787]	0.245
Baseline serum phosphate	3	0	−9.652 [−55.48, 36.08]	0.277

Abbreviations: BSAP, bone specific alkaline phosphatase; CI, confidence interval; CTX, C-terminal telopeptide of type 1 collagen; PINP, procollagen type I N-terminal propeptide; PTH, parathyroid hormone; SE, standard error

The predictors of the efficacy of ibandronate in improving BMD of the total hip in subjects with osteoporosis or decreased BMD (all conditions) were: Treatment duration from 1 to 5 years, increasing age, history of previous fractures, lower baseline T score of total hip, and higher baseline serum CTX (Table 1; Figs 2 and 3). Number of participants, gender, weight, height, BMI, baseline serum levels of 25-OH vitamin D, PTH, PINP, osteocalcin, calcium and phosphate did not show any significant relationship with the efficacy of ibandronate in improving total hip BMD in subjects with osteoporosis or decreased BMD.

In postmenopausal women with osteoporosis or decreased BMD, longer treatment duration (1 to 5 years), increasing age, lower body weight, increasing time since menopause, lower baseline T score of lumbar spine, lower baseline serum levels of 25-OH vitamin D, and higher baseline serum levels of CTX and BSAP predicted better efficacy of ibandronate in improving BMD of the lumbar spine (Table 2). In these women, increasing age, increasing time since menopause, lower BMI, lower baseline T score of total hip, and higher baseline serum levels of CTX predicted better efficacy of ibandronate in improving BMD of the total hip (Table 2).

**Table 2:** Predictors of the efficacy of ibandronate in improving BMD of the PMO patients and postmenopausal women with decreased BMD.

Variables	Datasets	tau^2^	Coefficient [95% CI]	P
**Lumbar Spine**				
Treatment duration	37	3.535	0.551 [−0.088, 1.189]	**0.089**
Number of participants	37	3.579	0.002 [−0.0005, 0.004]	0.122
Age	37	1.882	0.329 [0.214, 0.443]	**<0.0001**
Body weight	27	2.213	−0.294 [−0.470, −0.118]	**0.002**
Body height	24	1.38	−0.162 [−0.441, 0.116]	0.240
Body mass index	27	2.006	−0.019 [−0.557, 0.519]	0.943
History of fractures	19	2.124	0.008 [−0.025, 0.042]	0.614
Time since menopause	27	2.037	0.295 [0.190, 0.401]	**<0.0001**
Baseline T score (lumbar spine)	37	1.876	−1.341 [−1.824, −0.858]	**<0.0001**
Baseline serum vitamin D 25OH	24	2.808	−0.237 [−0.372, −0.1025]	**0.001**
Baseline serum PTH	11	6.047	−0.001 [−0.103, 1.004]	0.979
Baseline serum osteocalcin	19	4.992	−0.113 [−0.276, 0.050]	0.162
Baseline serum BSAP	14	3.025	0.037 [0.007, 0.067]	**0.019**
Baseline serum CTX	21	2.028	17.99 [11.14, 24.84]	**<0.0001**
**Total hip**				
Treatment duration	27	0.664	0.246 [−0.052, 0.546]	0.102
Number of participants	27	0.730	0.0005 [−0.0006, 0.0016]	0.363
Age	27	0.5147	0.129 [0.046, 0.212]	**0.004**
Body weight	19	0.259	−0.069 [−0.217, 0.079]	0.338
Body height	19	0.258	−0.075 [−0.233, 0.083]	0.332
Body mass index	20	0.4538	−0.289 [−0.628, 0.049]	**0.089**
History of fractures	14	0.628	0.013 [−0.007, 0.034]	0.195
Time since menopause	20	0.476	0.114 [0.046, 0.018]	**0.002**
Baseline T score (total hip)	18	0.741	−0.852 [−1.686, −0.018]	**0.046**
Baseline serum vitamin D 25OH	18	0.657	−0.050 [−0.139, 0.038]	0.245
Baseline serum PTH	8	0.451	0.0007 [−0.038, 0.04]	0.964
Baseline serum osteocalcin	15	0958	0.017 [−0.0718, 0.106]	0.686
Baseline serum BSAP	12	1.067	0.009 [−0.01, 0.029]	0.319
Baseline serum CTX	13	0.5014	6.019 [1.462, 10.577]	**0.014**

Abbreviations: BSAP, bone specific alkaline phosphatase; CI, confidence interval; CTX, C-terminal telopeptide of type 1 collagen; PINP, procollagen type I N-terminal propeptide; PMO, postmenopausal osteoporosis; PTH, parathyroid hormone; SE, standard error

In non-PMO study subject with osteoporosis or decreased BMD, longer treatment duration (1–5 years) and history of previous fractures were identified as the predictors of better ibandronate efficacy in improving the BMD of lumbar spine (Table 3). Less data were available for the metaregression analyses of other variables for non-PMO osteoporosis subjects and individuals with decreased BMD.

**Table 3:** Predictors of the efficacy of ibandronate in improving BMD of lumbar spine in non-PMO patients and individuals with decreased BMD.

Variables	Datasets	tau^2^	Coefficient [95% CI]	P
Treatment duration	13	21.46	3.488 [−0.215, 7.192]	**0.062**
Number of participants	13	30.01	0.0062 [−0.0216, 0.034]	0.632
Gender	12	27.93	−0.005 [−0.101, 0.091]	0.908
Age	12	27.6	0.064 [−0.265, 0.394]	0.674
Body mass index	8	16.48	0.116 [−1.907, 2.14]	0.893
History of previous fractures	8	9.59	0.096 [−0.017, 0.209]	**0.083**
Baseline T score (lumbar spine)	13	26.72	−1.791 [−5.19, 1.61]	0.217

Abbreviations: BMD, bone mineral density; CI, confidence interval; PMO, postmenopausal osteoporosis

## Discussion

In the present metaregression analyses, longer treatment duration from 1 to 5 years, increasing age, lower body weight, lower baseline T score, and higher baseline levels of CTX predicted higher ibandronate efficacy in improving BMD of the lumbar spine as well as of total hip. Lower baseline levels of vitamin D and higher baseline levels of BSAP predicted higher efficacy of ibandronate for lumbar spine only. In postmenopausal subjects with osteoporosis or decreased BMD, in addition to above-mentioned predictors, better efficacy of ibandronate was also associated with increasing time since menopause. There was a significant negative relationship between the baseline age and baseline T score of lumbar spine (r = −0.55; p<0.0001) as well as of total hip (r = −0.279; p = 0.049).

### Postmenopausal women with osteoporosis or decreased BMD

In postmenopausal subjects with osteoporosis or decreased BMD, longer treatment duration (from 1 to 5 years) was associated with higher ibandronate efficacy in improving lumbar spine and total hip BMD in the present study. These results support the predictive model of Mandema et al [61] in which non-linear least squares random effects metaregression analyses were utilized to predict the differences from placebo in percent changes from baseline in BMD at 12, 24, and 36 months after ibandronate and other related therapies.

We have found a significant negative relationship between baseline T-scores of lumbar spine as well as of total hip and the efficacy of ibandronate. Moreover, there was a significant negative relationship between baseline age of the subjects and T scores. This observation suggests that ibandronate treatment is more effective in subjects with more serious conditions of osteoporosis and that ibandronate is useful for long-term use. However, there can be a potential selection bias in the trials’ recruitment phase of the included studies as many other studies have reported a positive relationship between the start of any treatment for osteoporosis and T scores [62–65]. Moreover, diagnosis and treatment rates of osteoporosis are quite low [66,67].

Age is strongly associated with osteoporosis and fracture risk [68–70]. In this study we have found that both with increasing age and increasing time since menopause the efficacy of ibandronate increases in improving BMD of lumbar spine as well as total hip in postmenopausal subjects with osteoporosis or decreased BMD. However, in these subjects, the efficacy of ibandronate in improving BMD of both lumbar spine and total hip was inversely associated with baseline T scores. Thus, it seems plausible that ibandronate efficacy increases as the baseline T-score decreases and that age per se is not a good predictor of ibandronate efficacy.

Osteoclasts degrade type I collagen (one of the most abundant bone constituents) as a result CTX is released in the blood. Bisphosphonate mediated CTX decrease can be quantified as earlier as one week after the start of treatment [71]. In the present study, higher baseline serum CTX levels predicted higher efficacy of ibandronate in improving BMD of both lumbar spine and total hip, and after treatment, this association became negative (r = −0.736; p<0.0001 for lumbar spine and r = −0.5; p = 0.009 for total hip). There was also a significant positive association between age and baseline CTX levels (r = 0.583; p = 0.01) and a significant negative association between the percent change in CTX following ibandronate treatment and age (coefficient: −2.558; p = 0.005). Although, decrease in CTX can promote osteonecrosis of the jaw [69], the risk assessment of CTX mediated osteonecrosis of the jaw bone is controversial [72]. Overall safety profile of ibandronate is good and for some parameters better than that of other aminobisphosphonates [73].

Another finding of the present study is that there was a significant negative relationship between baseline 25-OH vitamin D and the efficacy of ibandronate in improving the BMD of lumbar spine but not of total hip. Moreover, there was a significant positive correlation between baseline vitamin D levels and baseline T score of lumbar spine (r = 0.505; p = 0.019) but not of total hip. Although, inadequate vitamin D levels are reported in individuals with hip fracture but fracture incidence itself can influence vitamin D levels [74–76]. There is evidence to suggest that vitamin D alone is less effective for hip bone strength e.g. in elderly women, one year treatment with calcium and vitamin D prevented hip BMD decrease but the effects were mainly in the women with below median baseline 25-OH vitamin D [77] and a meta-analysis of 53 trials (91,791 subjects) did not find vitamin D supplementation effective in preventing hip fractures [78]. Thus it may be possible that vitamin D have differential effects on lumbar spine and hip bones.

In the present study, lower BMI was a significant predictor of ibandronate treatment in improving total hip BMD in in postmenopausal subjects with osteoporosis or decreased BMD. Additionally, lower body weight was also predicting better ibandronate efficacy in improving lumbar spine BMD in overall population of the present study. Lower BMI is well-reported to be associated with higher fracture risk [79,80].

### Non-PMO subjects with osteoporosis or decreased BMD

Relatively less data were available to test explanatory variables against outcome variables in order to evaluate the predictors of ibandronate in improving lumbar spine BMD in non-postmenopausal subjects with osteoporosis or decreased BMD. Longer treatment duration (from 1 to 5 years) and history of previous fractures were positively associated with higher ibandronate efficacy in improving lumbar spine BMD in the present study.

Data were inadequate to evaluate the predictors of ibandronate in improving total hip BMD. Thus, with regards to non-postmenopausal subjects with osteoporosis or decreased BMD, outcomes remain inconclusive till the availability of adequate data in future trials.

## Conclusion

In individuals with osteoporosis, especially postmenopausal women with osteoporosis or decreased BMD, ibandronate therapy has been found more effective in more serious conditions and when applied for longer durations. In particular, longer treatment duration from 1 to 5 years, increasing age, lower baseline T score, lower body weight, and higher baselines serum CTX levels predicted better efficacy of ibandronate. Lower baseline vitamin D levels and higher serum BSAP levels predicted higher efficacy of ibandronate for lumbar spine but not for total hip.
